# One sixth of Amazonian tree diversity is dependent on river floodplains

**DOI:** 10.1038/s41559-024-02364-1

**Published:** 2024-03-11

**Authors:** John Ethan Householder, Florian Wittmann, Jochen Schöngart, Maria Teresa Fernandez Piedade, Wolfgang J. Junk, Edgardo Manuel Latrubesse, Adriano Costa Quaresma, Layon O. Demarchi, Guilherme de S. Lobo, Daniel P. P. de Aguiar, Rafael L. Assis, Aline Lopes, Pia Parolin, Iêda Leão do Amaral, Luiz de Souza Coelho, Francisca Dionízia de Almeida Matos, Diógenes de Andrade Lima Filho, Rafael P. Salomão, Carolina V. Castilho, Juan Ernesto Guevara-Andino, Marcelo de Jesus Veiga Carim, Oliver L. Phillips, Dairon Cárdenas López, William E. Magnusson, Daniel Sabatier, Juan David Cardenas Revilla, Jean-François Molino, Mariana Victória Irume, Maria Pires Martins, José Renan da Silva Guimarães, José Ferreira Ramos, Domingos de Jesus Rodrigues, Olaf S. Bánki, Carlos A. Peres, Nigel C. A. Pitman, Joseph E. Hawes, Everton José Almeida, Luciane Ferreira Barbosa, Larissa Cavalheiro, Márcia Cléia Vilela dos Santos, Bruno Garcia Luize, Evlyn Márcia Moraes de Leão Novo, Percy Núñez Vargas, Thiago Sanna Freire Silva, Eduardo Martins Venticinque, Angelo Gilberto Manzatto, Neidiane Farias Costa Reis, John Terborgh, Katia Regina Casula, Flávia R. C. Costa, Euridice N. Honorio Coronado, Abel Monteagudo Mendoza, Juan Carlos Montero, Ted R. Feldpausch, Gerardo A. Aymard C, Chris Baraloto, Nicolás Castaño Arboleda, Julien Engel, Pascal Petronelli, Charles Eugene Zartman, Timothy J. Killeen, Lorena Maniguaje Rincón, Beatriz S. Marimon, Ben Hur Marimon-Junior, Juliana Schietti, Thaiane R. Sousa, Rodolfo Vasquez, Bonifacio Mostacedo, Dário Dantas do Amaral, Hernán Castellanos, Marcelo Brilhante de Medeiros, Marcelo Fragomeni Simon, Ana Andrade, José Luís Camargo, William F. Laurance, Susan G. W. Laurance, Emanuelle de Sousa Farias, Maria Aparecida Lopes, José Leonardo Lima Magalhães, Henrique Eduardo Mendonça Nascimento, Helder Lima de Queiroz, Roel Brienen, Pablo R. Stevenson, Alejandro Araujo-Murakami, Tim R. Baker, Bruno Barçante Ladvocat Cintra, Yuri Oliveira Feitosa, Hugo F. Mogollón, Janaína Costa Noronha, Flávia Rodrigues Barbosa, Rainiellen de Sá Carpanedo, Joost F. Duivenvoorden, Miles R. Silman, Leandro Valle Ferreira, Carolina Levis, José Rafael Lozada, James A. Comiskey, Freddie C. Draper, José Julio de Toledo, Gabriel Damasco, Nállarett Dávila, Roosevelt García-Villacorta, Alberto Vicentini, Fernando Cornejo Valverde, Alfonso Alonso, Luzmila Arroyo, Francisco Dallmeier, Vitor H. F. Gomes, Eliana M. Jimenez, David Neill, Maria Cristina Peñuela Mora, Fernanda Antunes Carvalho, Fernanda Coelho de Souza, Kenneth J. Feeley, Rogerio Gribel, Marcelo Petratti Pansonato, Marcos Ríos Paredes, Jos Barlow, Erika Berenguer, Kyle G. Dexter, Joice Ferreira, Paul V. A. Fine, Marcelino Carneiro Guedes, Isau Huamantupa-Chuquimaco, Juan Carlos Licona, Toby Pennington, Boris Eduardo Villa Zegarra, Vincent Antoine Vos, Carlos Cerón, Émile Fonty, Terry W. Henkel, Paul Maas, Edwin Pos, Marcos Silveira, Juliana Stropp, Raquel Thomas, Doug Daly, William Milliken, Guido Pardo Molina, Ima Célia Guimarães Vieira, Bianca Weiss Albuquerque, Wegliane Campelo, Thaise Emilio, Alfredo Fuentes, Bente Klitgaard, José Luis Marcelo Pena, Priscila F. Souza, J. Sebastián Tello, Corine Vriesendorp, Jerome Chave, Anthony Di Fiore, Renato Richard Hilário, Luciana de Oliveira Pereira, Juan Fernando Phillips, Gonzalo Rivas-Torres, Tinde R. van Andel, Patricio von Hildebrand, William Balee, Edelcilio Marques Barbosa, Luiz Carlos de Matos Bonates, Hilda Paulette Dávila Doza, Ricardo Zárate Gómez, Therany Gonzales, George Pepe Gallardo Gonzales, Bruce Hoffman, André Braga Junqueira, Yadvinder Malhi, Ires Paula de Andrade Miranda, Linder Felipe Mozombite-Pinto, Adriana Prieto, Agustín Rudas, Ademir R. Ruschel, Natalino Silva, César I. A. Vela, Stanford Zent, Egleé L. Zent, Angela Cano, Yrma Andreina Carrero Márquez, Diego F. Correa, Janaina Barbosa Pedrosa Costa, Bernardo Monteiro Flores, David Galbraith, Milena Holmgren, Michelle Kalamandeen, Marcelo Trindade Nascimento, Alexandre A. Oliveira, Hirma Ramirez-Angulo, Maira Rocha, Veridiana Vizoni Scudeller, Rodrigo Sierra, Milton Tirado, Maria Natalia Umaña, Geertje van der Heijden, Emilio Vilanova Torre, Manuel Augusto Ahuite Reategui, Cláudia Baider, Henrik Balslev, Sasha Cárdenas, Luisa Fernanda Casas, William Farfan-Rios, Cid Ferreira, Reynaldo Linares-Palomino, Casimiro Mendoza, Italo Mesones, Germaine Alexander Parada, Armando Torres-Lezama, Ligia Estela Urrego Giraldo, Daniel Villarroel, Roderick Zagt, Miguel N. Alexiades, Edmar Almeida de Oliveira, Karina Garcia-Cabrera, Lionel Hernandez, Walter Palacios Cuenca, Susamar Pansini, Daniela Pauletto, Freddy Ramirez Arevalo, Adeilza Felipe Sampaio, Elvis H. Valderrama Sandoval, Luis Valenzuela Gamarra, Hans ter Steege

**Affiliations:** 1 Wetland Department, Institute of Geography and Geoecology, Karlsruhe Institute of Technology, Rastatt, Germany; 2 Ecology, Monitoring and Sustainable Use of Wetlands, Instituto Nacional de Pesquisas da Amazônia, Manaus, Brazil; 3 National Institute for Science and Technology of Wetlands, Federal University of Mato Grosso, Cuiabá, Brazil; 4 Environmental Sciences Graduate Program-CIAMB, Federal University of Goiás, Goiânia, Brazil; 5 Procuradoria-Geral de Justiça, Ministério Público do Estado do Amazonas, Manaus, Brazil; 6 Coordenação de Dinâmica Ambiental, Instituto Nacional de Pesquisas da Amazônia, Manaus, Brazil; 7 Biodiversity and Ecosystem Services, Instituto Tecnológico Vale, Belém, Brazil; 8 Department of Ecology, Institute of Biological Sciences, University of Brasilia, Brasilia, Brazil; 9 Biocentre Klein Flottbek and Botanical Gardens, University of Hamburg, Hamburg, Germany; 10 Coordenação de Biodiversidade, Instituto Nacional de Pesquisas da Amazônia, Manaus, Brazil; 11 Programa Professor Visitante Nacional Sênior na Amazônia—CAPES, Universidade Federal Rural da Amazônia, Belém, Brazil; 12 Coordenação de Botânica, Museu Paraense Emílio Goeldi, Belém, Brazil; 13 Centro de Pesquisa Agroflorestal de Roraima, Embrapa Roraima, Boa Vista, Brazil; 14 Grupo de Investigación en Ecología y Evolución en los Trópicos, Universidad de las Américas, Quito, Ecuador; 15 Keller Science Action Center, the Field Museum, Chicago, IL USA; 16 Departamento de Botânica, Instituto de Pesquisas Científicas e Tecnológicas do Amapá, Macapá, Brazil; 17 School of Geography, University of Leeds, Leeds, UK; 18 Herbario Amazónico Colombiano, Instituto SINCHI, Bogotá, Colombia; 19 Coordenação de Pesquisas em Ecologia, Instituto Nacional de Pesquisas da Amazônia, Manaus, Brazil; 20 AMAP, IRD, Cirad, CNRS, INRAE, Université de Montpellier, Montpellier, France; 21 Amcel Amapá Florestal e Celulose S.A, Santana, Brazil; 22 ICNHS, Federal University of Mato Grosso, Sinop, Brazil; 23 Catalogue of Life, Leiden, the Netherlands; 24 School of Environmental Sciences, University of East Anglia, Norwich, UK; 25 Science and Education, the Field Museum, Chicago, IL USA; 26 Institute of Science and Environment, University of Cumbria, Ambleside, UK; 27 ICNHS, Universidade Federal de Mato Grosso, Sinop, Brazil; 28 Departamento de Biologia Vegetal, Instituto de Biologia, Universidade Estadual de Campinas, Campinas, Brazil; 29 Divisao de Sensoriamento Remoto, Instituto Nacional de Pesquisas Espaciais, São José dos Campos, Brazil; 30 Herbario Vargas, Universidad Nacional de San Antonio Abad del Cusco, Cusco, Peru; 31 Biological and Environmental Sciences, University of Stirling, Stirling, UK; 32 Centro de Biociências, Departamento de Ecologia, Universidade Federal do Rio Grande do Norte, Natal, Brazil; 33 Departamento de Biologia, Universidade Federal de Rondônia, Porto Velho, Brazil; 34 Programa de Pós-Graduação em Biodiversidade e Biotecnologia PPG- Bionorte, Universidade Federal de Rondônia, Porto Velho, Brazil; 35 Department of Biology and Florida Museum of Natural History, University of Florida, Gainesville, FL USA; 36 Centre for Tropical Environmental and Sustainability Science and College of Science and Engineering, James Cook University, Cairns, Queensland Australia; 37 Instituto de Investigaciones de la Amazonía Peruana, Iquitos, Peru; 38 School of Geography and Sustainable Development, University of St Andrews, St Andrews, UK; 39 Jardín Botánico de Missouri, Oxapampa, Peru; 40 Instituto Boliviano de Investigacion Forestal, Santa Cruz, Bolivia; 41 Geography, College of Life and Environmental Sciences, University of Exeter, Exeter, UK; 42 Programa de Ciencias del Agro y el Mar, Herbario Universitario (PORT), UNELLEZ-Guanare, Guanare, Venezuela; 43 International Center for Tropical Botany Department of Biological Sciences, Florida International University, Miami, FL USA; 44 Cirad UMR Ecofog, AgrosParisTech, CNRS, INRAE, Université de Guyane, Kourou, France; 45 Agteca-Amazonica, Santa Cruz, Bolivia; 46 Programa de Pós-Graduação em Ecologia e Conservação, Universidade do Estado de Mato Grosso, Nova Xavantina, Brazil; 47 Programa de Pós-Graduação em Ecologia, Instituto Nacional de Pesquisas da Amazônia, Manaus, Brazil; 48 Facultad de Ciencias Agrícolas, Universidad Autónoma Gabriel René Moreno, Santa Cruz, Bolivia; 49 Centro de Investigaciones Ecológicas de Guayana, Universidad Nacional Experimental de Guayana, Bolivar, Venezuela; 50 Embrapa Recursos Genéticos e Biotecnologia, Parque Estação Biológica, Prédio da Botânica e Ecologia, Brasilia, Brazil; 51 Projeto Dinâmica Biológica de Fragmentos Florestais, Instituto Nacional de Pesquisas da Amazônia, Manaus, Brazil; 52 Laboratório de Ecologia de Doenças Transmissíveis da Amazônia, Instituto Leônidas e Maria Deane, Fiocruz, Manaus, Brazil; 53 Programa de Pós-graduação em Biodiversidade e Saúde, Instituto Oswaldo Cruz, Rio de Janeiro, Brazil; 54 Instituto de Ciências Biológicas, Universidade Federal do Pará, Belém, Brazil; 55 Programa de Pós-Graduação em Ecologia, Universidade Federal do Pará, Belém, Brazil; 56 Empresa Brasileira de Pesquisa Agropecuária, Embrapa Amazônia Oriental, Belém, Brazil; 57 Diretoria Técnico-Científica, Instituto de Desenvolvimento Sustentável Mamirauá, Tefé, Brazil; 58 Laboratorio de Ecología de Bosques Tropicales y Primatología, Universidad de los Andes, Bogotá, Colombia; 59 Museo de Historia Natural Noel Kempff Mercado, Universidad Autónoma Gabriel Rene Moreno, Santa Cruz, Bolivia; 60 Birmingham Institute for Forest Research, University of Birmingham, Birmingham, UK; 61 Programa de Pós-Graduação em Biologia (Botânica), Instituto Nacional de Pesquisas da Amazônia, Manaus, Brazil; 62 Endangered Species Coalition, Silver Spring, MD USA; 63 Institute of Biodiversity and Ecosystem Dynamics, University of Amsterdam, Amsterdam, the Netherlands; 64 Biology Department and Center for Energy, Environment and Sustainability, Wake Forest University, Winston Salem, NC USA; 65 Graduate Program in Ecology, Federal University of Santa Catarina, Florianópolis, Brazil; 66 Facultad de Ciencias Forestales y Ambientales, Instituto de Investigaciones para el Desarrollo Forestal, Universidad de los Andes, Mérida, Venezuela; 67 Inventory and Monitoring Program, National Park Service, Fredericksburg, VA USA; 68 Center for Conservation and Sustainability, Smithsonian Conservation Biology Institute, Washington, DC USA; 69 Department of Geography and Planning, University of Liverpool, Liverpool, UK; 70 Ciências Ambientais, Universidade Federal do Amapá, Macapá, Brazil; 71 Gothenburg Global Biodiversity Centre, University of Gothenburg, Gothenburg, Sweden; 72 Programa Restauración de Ecosistemas, Centro de Innovación Científica Amazónica, Tambopata, Peru; 73 Peruvian Center for Biodiversity and Conservation, Iquitos, Peru; 74 Andes to Amazon Biodiversity Program, Madre de Dios, Peru; 75 Escola de Negócios Tecnologia e Inovação, Centro Universitário do Pará, Belém, Brazil; 76 Environmental Science Program, Geosciences Department, Universidade Federal do Pará, Belém, Brazil; 77 Grupo de Ecología y Conservación de Fauna y Flora Silvestre, Instituto Amazónico de Investigaciones Imani, Universidad Nacional de Colombia sede Amazonia, Leticia, Colombia; 78 Universidad Estatal Amazónica, Puyo, Ecuador; 79 Universidad Regional Amazónica IKIAM, Tena, Ecuador; 80 Instituto de Ciências Biológicas, Departamento de Genética, Ecologia e Evolução, Universidade Federal de Minas Gerais, Belo Horizonte, Brazil; 81 Department of Biology, University of Miami, Coral Gables, FL USA; 82 Fairchild Tropical Botanic Garden, Coral Gables, FL USA; 83 Instituto de Biociências, Departamento de Ecologia, Universidade de Sao Paulo, São Paulo, Brazil; 84 Servicios de Biodiversidad EIRL, Iquitos, Peru; 85 Lancaster Environment Centre, Lancaster University, Lancaster, UK; 86 Environmental Change Institute, University of Oxford, Oxford, UK; 87 School of Geosciences, University of Edinburgh, Edinburgh, UK; 88 Tropical Diversity Section, Royal Botanic Garden Edinburgh, Edinburgh, UK; 89 Department of Integrative Biology, University of California, Berkeley, Berkeley, CA USA; 90 Empresa Brasileira de Pesquisa Agropecuária, Embrapa Amapá, Macapá, Brazil; 91 Herbario HAG, Universidad Nacional Amazónica de Madre de Dios, Puerto Maldonado, Peru; 92 Direccíon de Evaluación Forestal y de Fauna Silvestre, Magdalena del Mar, Peru; 93 Instituto de Investigaciones Forestales de la Amazonía, Universidad Autónoma del Beni José Ballivián, Riberalta, Bolivia; 94 Escuela de Biología Herbario Alfredo Paredes, Universidad Central, Quito, Ecuador; 95 Office national des forêts, Direction régionale de la Guyane, Cayenne, French Guiana; 96 Department of Biological Sciences, California State Polytechnic University, Arcata, CA USA; 97 Naturalis Biodiversity Center, Leiden, the Netherlands; 98 Quantitative Biodiversity Dynamics, Utrecht University, Utrecht, the Netherlands; 99 Utrecht University Botanic Gardens, Utrecht, the Netherlands; 100 Centro de Ciências Biológicas e da Natureza, Universidade Federal do Acre, Rio Branco, Brazil; 101 Biogeography Department, Trier University, Trier, Germany; 102 Iwokrama International Centre for Rain Forest Conservation and Development, Georgetown, Guyana; 103 New York Botanical Garden, Bronx, New York, NY USA; 104 Department for Ecosystem Stewardship, Royal Botanic Gardens, Kew, Richmond, UK; 105 Herbario Nacional de Bolivia, Universitario UMSA, La Paz, Bolivia; 106 Center for Conservation and Sustainable Development, Missouri Botanical Garden, St. Louis, MO USA; 107 Department for Accelerated Taxonomy, Royal Botanic Gardens, Kew, Richmond, UK; 108 Universidad Nacional de Jaén, Cajamarca, Peru; 109 Laboratoire Evolution et Diversité Biologique, CNRS and Université Paul Sabatier, UMR 5174 EDB, Toulouse, France; 110 Department of Anthropology, University of Texas at Austin, Austin, TX USA; 111 Estación de Biodiversidad Tiputini, Colegio de Ciencias Biológicas y Ambientales, Universidad San Francisco de Quito, Quito, Ecuador; 112 Fundación Puerto Rastrojo, Bogotá, Colombia; 113 Department of Wildlife Ecology and Conservation, University of Florida, Gainesville, FL USA; 114 Biosystematics Group, Wageningen University, Wageningen, the Netherlands; 115 Fundación Estación de Biología, Bogotá, Colombia; 116 Department of Anthropology, Tulane University, New Orleans, LA USA; 117 PROTERRA, Instituto de Investigaciones de la Amazonía Peruana, Iquitos, Peru; 118 ACEER Foundation, Puerto Maldonado, Peru; 119 Amazon Conservation Team, Arlington, VA USA; 120 Institut de Ciència i Tecnologia Ambientals, Universitat Autònoma de Barcelona, Barcelona, Spain; 121 Environmental Change Institute, Oxford University Centre for the Environment, Oxford, UK; 122 Instituto de Ciencias Naturales, Universidad Nacional de Colombia, Bogotá, Colombia; 123 Instituto de Ciência Agrárias, Universidade Federal Rural da Amazônia, Belém, Brazil; 124 Escuela Profesional de Ingeniería Forestal, Universidad Nacional de San Antonio Abad del Cusco, Puerto Maldonado, Peru; 125 Laboratory of Human Ecology, Instituto Venezolano de Investigaciones Científicas, Caracas, Venezuela; 126 Cambridge University Botanic Garden, Cambridge University, Cambridge, UK; 127 Programa de Maestria de Manejo de Bosques, Universidad de los Andes, Mérida, Venezuela; 128 Centre for Biodiversity and Conservation Science, University of Queensland, Brisbane, Queensland Australia; 129 Resource Ecology Group, Wageningen University & Research, Wageningen, the Netherlands; 130 School of Earth, Environment and Society, McMaster University, Hamilton, Ontario Canada; 131 Laboratório de Ciências Ambientais, Universidade Estadual do Norte Fluminense, Campos dos Goytacazes, Brazil; 132 Instituto de Investigaciones para el Desarrollo Forestal, Universidad de los Andes, Mérida, Venezuela; 133 Departamento de Biologia, Instituto de Ciências Biológicas, Universidade Federal do Amazonas, Manaus, Brazil; 134 GeoIS, Quito, Ecuador; 135 Department of Ecology and Evolutionary Biology, University of Michigan, Ann Arbor, MI USA; 136 Faculty of Social Sciences, University of Nottingham, Nottingham, UK; 137 Wildlife Conservation Society, New York, NY USA; 138 Medio Ambiente, PLUSPRETOL, Iquitos, Peru; 139 Mauritius Herbarium, Agricultural Services, Ministry of Agro-Industry and Food Security, Moka, Mauritius; 140 Department of Biology, Aarhus University, Aarhus, Denmark; 141 Escuela de Ciencias Forestales, Universidad Mayor de San Simon, Cochabamba, Bolivia; 142 FOMABO, Manejo Forestal en las Tierras Tropicales de Bolivia, Cochabamba, Bolivia; 143 Departamento de Ciencias Forestales, Universidad Nacional de Colombia, Medellín, Colombia; 144 Fundación Amigos de la Naturaleza, Santa Cruz, Bolivia; 145 Tropenbos International, Ede, the Netherlands; 146 School of Anthropology and Conservation, University of Kent, Canterbury, UK; 147 Herbario Nacional del Ecuador, Universidad Técnica del Norte, Quito, Ecuador; 148 Instituto de Biodiversidade e Florestas, Universidade Federal do Oeste do Pará, Santarém, Brazil; 149 Facultad de Biologia, Universidad Nacional de la Amazonia Peruana, Iquitos, Peru; 150 Department of Biology, University of Missouri, St. Louis, MO USA

**Keywords:** Biodiversity, Community ecology

## Abstract

Amazonia’s floodplain system is the largest and most biodiverse on Earth. Although forests are crucial to the ecological integrity of floodplains, our understanding of their species composition and how this may differ from surrounding forest types is still far too limited, particularly as changing inundation regimes begin to reshape floodplain tree communities and the critical ecosystem functions they underpin. Here we address this gap by taking a spatially explicit look at Amazonia-wide patterns of tree-species turnover and ecological specialization of the region’s floodplain forests. We show that the majority of Amazonian tree species can inhabit floodplains, and about a sixth of Amazonian tree diversity is ecologically specialized on floodplains. The degree of specialization in floodplain communities is driven by regional flood patterns, with the most compositionally differentiated floodplain forests located centrally within the fluvial network and contingent on the most extraordinary flood magnitudes regionally. Our results provide a spatially explicit view of ecological specialization of floodplain forest communities and expose the need for whole-basin hydrological integrity to protect the Amazon’s tree diversity and its function.

## Abstract (web summary)

Analysing >1,700 inventory plots from the Amazon Tree Diversity Network, the authors show that the majority of Amazon tree species can occupy floodplains and that patterns of species turnover are closely linked to regional flood patterns.

## Main

Amazonia’s floodplain forests border the rivers that collectively make up our planet’s largest fluvial system and underpin crucial aspects of floodplain ecosystem function^1^. But how and why are the Amazon’s floodplain tree communities distinct from surrounding forests? These questions have long intrigued ecologists because they get to the heart of what floodplain forests mean for the maintenance of tree diversity^2–4^, population regulation^5,6^ and speciation^7–10^. These questions are vital for conservation planning too. Natural flood regimes are a principal driver of growth, phenology and life cycles of floodplain trees^1^ but are becoming increasingly altered by proliferating hydroelectric dams, changing rainfall patterns and deforestation^11–13^. These changes threaten to reshape floodplain tree assemblies in ways that imperil both biodiversity and fundamental ecosystem functions^14–16^. For example, compositional changes to floodplain forests are expected to affect crucial fish–tree interactions that sustain aquatic trophic webs, with unknown consequences for productive fisheries on which the livelihoods of Amazonian peoples depend^17^. Given the evidence that human interventions in the Amazon’s hydrological system are disruptive to floodplain tree communities and propagate over large spatial scales^11–13^, our understanding of the species composition of floodplain forests and the extent to which they differ from surrounding forest types is still too fragmented^18^, coming mostly from studies with limited spatial extents (but see refs. ^7,9,19^). Amid the growing pace and scope of hydrological threats, there is a pressing need for an integrated, system-wide approach that can guide floodplain conservation strategies and identify potential vulnerabilities in spatially explicit ways.

Central to this aim are two concepts that together capture essential information about linkages between species and their environment. The first is habitat specialization, which measures the restriction of a tree species’ distribution to particular environments (here, to floodplains). It sheds light on species adaptation and ecological function and can be a key indicator of species vulnerability to environmental change^20^. The second is species turnover—here as a measure of the level of compositional differentiation between floodplain and adjacent terra firme forest—which reflects the extent to which species distributions are constrained by habitat, and thus how tree diversity is spatially organized on landscapes^21^. Because both concepts link species to their environment and capture the component of forest diversity that is unique to floodplains, they offer key insights into how floodplains regulate species populations and maintain tree diversity on Amazonian landscapes as well as related challenges for floodplain conservation^21^. Our understanding of the geographic and environmental patterning of habitat specialization and species turnover remains inadequate in the context of floodplain forests, but it will be a crucial feature of our ability to safeguard the biodiversity of future floodplain ecosystems and ensure their ecological functioning amid growing hydrological risks.

## Dataset and approach

We examined Amazon-wide patterns of floodplain tree specialization and species turnover by analysing a uniquely suited dataset of 1,705 mostly 1 ha tree inventory plots, with information on species composition and abundances, from the Amazon Tree Diversity Network (ATDN)^22^. This includes both floodplain (*n* = 455) and terra firme (*n* = 1,250) sites, extending across most of the Amazon region and enabling us to assess species turnover and specialization across greater spatial, environmental and floristic heterogeneity than previously possible. We first used a spatially explicit approach to examine Amazon-wide patterns of tree species turnover between seasonal floodplain forests and surrounding, non-flooded terra firme. We found clear geographic patterning and subsequently considered a suspected driver, regional differences in flooding^7,9,23,24^. Second, we assessed species-specific habitat patterns with tests of floodplain specialization for over 1,600 tree species, examining the local abundance patterns of floodplain specialists. Finally, we considered how our findings contribute to discussions of floodplain biodiversity conservation in the face of systemic hydrological change.

## Results and discussion

### Geographic patterning of species turnover

**Extended Data Fig. 1:**
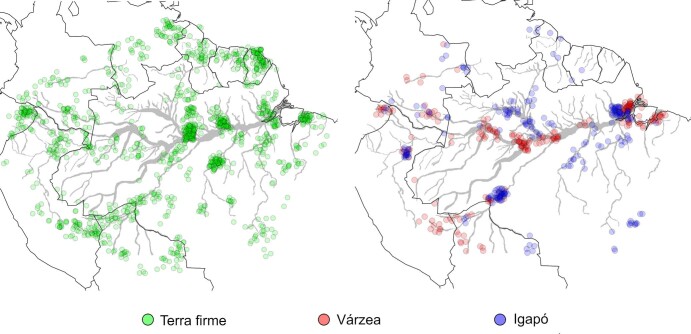
Distribution of inventory data used to create habitat-specific compositional grids. Sampled sites include 1,705 mostly 1-ha tree inventory plots with full information on species composition and abundances. Plots were classified as terra firme (n = 1,250, 73%), *várzea* (n = 271, 16%), or *igapó* (n = 184, 11%), following habitat designations of ATDN contributors.

**Extended Data Fig. 2:**
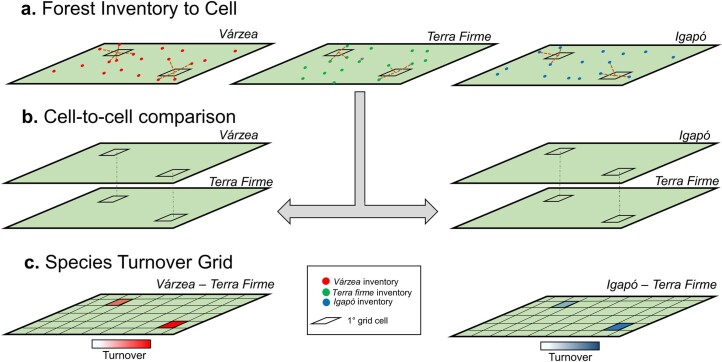
Schematic of the methods used to compare floodplain and terra firme tree compositions, illustrated for two grid cells. (**a**) Forest plot inventories (colored dots) were separated into *várzea*, *igapó* and terra firme categories, and species abundance information for separate *várzea*, *igapó* and terra firme grids was calculated at each 1-degree cell (only two shown), using distance-weighted interpolations of inventory plot data from an approximately 300 km circular window (red lines). (**b**) Floodplain and terra firme grids were overlaid and species turnover computed at analogous (vertically overlapping) cells. (**c**) Spatially-continuous grids of species turnover for *várzea*-terra firme and *igapó*-terra firme comparisons. The number of cells where species turnover is calculated depends on the spatial distribution of floodplain inventories and how it overlaps with terra firme inventories. This included 301 cells and 347 cells for *várzea-*terra firme and *igapó-*terra firme comparisons, respectively. In an alternative procedure of calculating species turnover, the interpolation step was excluded and cell compositional data was pooled only from plots located inside cells. For this second approach, the resulting number of cells where species turnover was calculated was 25 and 22 for *várzea-*terra firme and *igapó-*terra firme comparisons, respectively.

To assess spatial variation in species turnover between floodplain and terra firme forests, we first used plot data (Extended Data Fig. 1 and Supplementary Table 1) to produce separate 1° compositional grids for three habitats: (1) terra firme is the predominant Amazonian forest type, without seasonal river flooding; (2) *várzea* is seasonally flooded forest that borders white-water rivers, characterized by their laterally migrating channels, high sediment loads and relatively fertile substrates of Andean origin^25^; and (3) *igapó* is seasonally flooded forest that lines black- and clear-water rivers with relatively stable channels, low sediment loads and nutrient-poor substrates^25^. The two floodplain classes capture important differences in limnology and chemistry that can strongly influence forest composition and therefore species turnover with adjacent terra firme^25^. The distinction of floodplains is recognizable in the field by the colour of the river water, and we used the reported field designations of ATDN data contributors. We populated cell compositional data for each habitat grid by spatially interpolating habitat-specific species densities (with distance decay) from inventories located within a ~300 km circular window. Spatially continuous maps of landscape-scale species turnover for *várzea*–terra firme and *igapó*–terra firme comparisons were then produced by measuring differences in tree species compositions at analogous cells of overlapping floodplain and terra firme compositional grids (Extended Data Fig. 2).

**Fig. 1:**
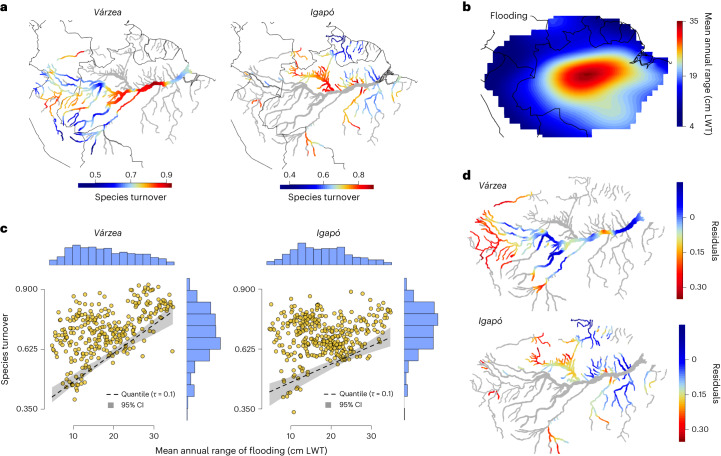
Broad-scale geographic and environmental patterning of species turnover across floodplain and adjacent terra firme forest habitats, for *várzea*–terra firme and *igapó*–terra firme comparisons. **a**, Spatial patterns of species turnover for *várzea* and *igapó*, showing a concentration of high species turnover located centrally within the fluvial network. Grey rivers are masked out because they either correspond to a different floodplain habitat or did not meet minimum sampling criteria for analysis. **b**, Regional differences in seasonal flooding are described as an annual flood wave that originates in Andean headwaters, peaks in central Amazonia and dissipates near the Amazon mouth. Floodplains positioned at the peak of this flood wave are seasonally inundated by the highest-amplitude and longest-lasting floods. LWT, land water thickness. **c**, Patterning of species turnover of *várzea* and *igapó* with surrounding terra firme along the flood wave. The black dashed line shows the lower bound of species turnover with flooding, assessed with quantile regression at *τ* = 0.1. **d**, Mapped residuals from quantile regression modelling for *várzea* and *igapó*. Throughout much of western Amazonia, species turnover is relatively higher than expected given the lower flooding implied by its headwater position on the flood wave.

The resulting maps reveal striking spatial patterning, with a distinctive nucleus of high species turnover emerging in central Amazonia (Fig. 1a). The high species turnover implies that populations of tree species are more strongly constrained by habitat in this central region than elsewhere. Outside this core region, *várzea*s have a secondary concentration of high species turnover in the west, although most other peripheral regions tend to show lower levels of species turnover that suggest a greater degree of species spillover across floodplain and terra firme habitats that homogenizes their tree communities to a larger extent.

### Patterning of species turnover with the Amazon flood wave

Flooding is a principal driver of environmental heterogeneity with important implications for forest composition^1,7,18^. The magnitude of flooding at local scales is strongly governed by the position of floodplains along the Amazon flood wave—a seasonal, moving mass of water that initiates as orographic rainfall feeding headwater rivers with short and sporadic flooding; propagates towards major collecting channels with larger-amplitude, unimodal flood pulses; and finally dissipates further downstream into the tidally influenced river estuary region^1^. We described the flood wave using spatiotemporal data on Terrestrial Water Storage (TWS) from NASA’s GRACE Tellus (GRC-Tellus) satellite^26^, estimating at every grid cell the mean annual magnitude of seasonal changes in the mass of surface flood waters (cm of land water thickness), averaged over a 15-year period (2002–2017) (Fig. 1b). Central Amazonian floodplain forests positioned at the peak of this flood wave annually experience severe seasonal flood amplitudes surpassing 8 m and inundation periods lasting for most of the year. Floodplains peripheral to this central region are located at low positions on the flood wave and tend to experience seasonal flooding that is less severe.

We found that both *várzea* and *igapó* floodplains located at the peak of the flood wave (those experiencing larger flood magnitudes) always had high species turnover with surrounding terra firme, suggesting that flooding is the primary factor limiting the level of compositional overlap with terra firme. However, floodplains with lower flooding did not always have low species turnover; indeed, many maintained high species turnover (Fig. 1c). The resulting triangular pattern suggested that while flooding may set the minimum level of species turnover, above these minima other (unmeasured) factors may interact with flooding to maintain compositional differences. With potentially many interacting factors, standard regression methods are not capable of detecting appropriate relationships^27^, so we employed quantile regression to model the lower bounds of species turnover (the tenth percentile of species turnover conditional on flooding). An added benefit is that by describing the lower bounds of species turnover, model residuals more appropriately reflect the summed effect of possible interacting factors, permitting us to investigate their geographic patterns without prior knowledge of what they might be^27,28^.

**Extended Data Fig. 3:**
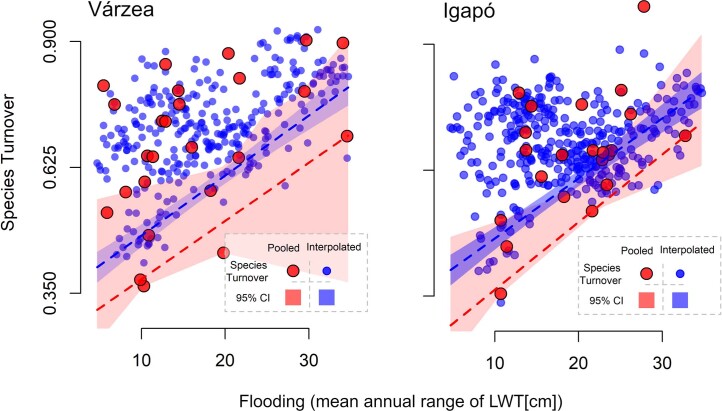
Comparison of flooding relationships with species turnover using two alternative procedures for populating cell compositional data. While interpolating species abundances maximizes the number of cells where species turnover can be calculated, it introduces spatial autocorrelation. On the other hand, pooling inventories within grid cells reduces the number of cells where species turnover can be calculated, but it maintains spatial independence among cells. We compared both methods to assess the robustness of our results to spatial dependencies. For the approach based on pooling, species cell abundance information was pooled only from inventories located inside individual grid cells, rather than interpolated from inventories from a larger 300 km circular window, in order to avoid residual spatial autocorrelation. Quantile regression slopes (at tau = 0.1) and their 95% confidences intervals are shown for *várzea*- and *igapó*-terra firme. The lower bounds of *várzea-*terra firme species turnover with flooding are statistically equivalent between pooled compositional data (slope ± 95% CI = 1.29 × 10^−2^ ± 1.21 × 10^−2^, t = 2.20, n = 25, p = 0.038) and interpolated data (slope ± 95% CI = 1.21 × 10^−2^ ± 2.48 × 10^−3^, t = 9.57, n = 301, p < 0.001). The lower bounds for *igapó-*terra firme are likewise similar between pooled (slope ± 95% CI = 1.54 × 10^−2^ ± 1.19 × 10^−2^, t = 2.66, n = 22, p = 0.015) and interpolated methods (slope ± 95% CI = 1.05 × 10^−2^ ± 2.60 × 10^−3^, t = 7.86, n = 347, p < 0.001). Slopes from all comparisons were significant (p < 0.05) and had overlapping 95% confidence intervals.

We found that the lower bounds of species turnover with flooding were statistically indistinguishable for both *várzea* and *igapó* floodplains, suggesting that the effect of flooding on species turnover is similar in both floodplain habitats (*várzea* slope ± 95% confidence interval (CI), 1.21 × 10^−2^ ± 2.48 × 10^−3^; *t* = 9.57; *n* = 301; *P* < 0.001) (*igapó* slope ± 95% CI, 1.05 × 10^−2^ ± 2.60 × 10^−3^; *t* = 7.86; *n* = 347; *P* < 0.001). Some residual spatial autocorrelation in both the *várzea* and *igapó* models (assessed with Moran’s *I* at *P* < 0.01) raised questions about the robustness of the observed relationships. This was expected because of the spatial dependencies built into the compositional response variable from interpolating species abundances, so we double-checked the observed relationships between flooding and species turnover using an alternative procedure for populating grid cells with plausible communities that did not involve spatial interpolation. Rather, we pooled species abundance information for each cell only from plot inventories directly located within that cell. This alternative procedure retains only well-sampled cells containing multiple floodplain and terra firme inventories but ensures cell-wise spatial independence of species abundance data. Despite the methodological differences, the relationships between species turnover and flooding were similar (*várzea*, 1.29 × 10^−2^ ± 1.21 × 10^−2^, *t* = 2.20, *n* = 25, *P* = 0.038; *igapó*, 1.54 × 10^−2^ ± 1.19 × 10^−2^, *t* = 2.66, *n* = 22, *P* = 0.015) (Extended Data Fig. 3). The patterns of species turnover with flooding therefore appear robust among methodological choices and similar across the two major floodplain types.

Geographic patterns of model residuals spotlighted a large area of northwestern Amazonia (particularly for *várzea*) with elevated residuals, implying that species turnover with surrounding terra firme was higher than expected given the region’s limited flooding and peripheral position far from the peak of the flood wave (Fig. 1d). High compositional differences may be maintained by one or more unmeasured factors that interact with flooding, or, alternatively, our estimate of the broad-scale flood wave failed to capture local but nonetheless important patterns of flooding that contribute to species turnover. For example, rivers in this area drain vast catchments exposed to some of the highest annual precipitation rates in Amazonia, which may influence local flooding patterns in ways that maintain compositional differences with surrounding terra firme (we explore this further in Supplementary Discussion 1, Supplementary Fig. 1 and Supplementary Table 2).

### Identifying floodplain specialist tree species

Differences in composition between floodplain and terra firme forests imply that many species may have narrow preferences for one or the other habitat. To better identify these specialized species, we employed association tests, which measure the strength of habitat preference for each species independently. We ran tests for a subset of 1,666 relatively well-sampled tree species (those with at least 20 occurrences in the plot network) using species-specific abundance information from plot inventories to test for non-random habitat selection for floodplains or terra firme. Specifically, we used a correlation index because absences outside a target habitat are also taken into account^29^, permitting us to categorize each species as a floodplain specialist, a terra firme specialist or a habitat generalist and subsequently examine each group’s relative abundance and richness in relation to species turnover.

Of the 1,666 species, a clear majority (1,450 species, 87%) occurred in floodplains at least once, and all had at least one occurrence in terra firme. Many tree species can therefore inhabit floodplains at least sporadically. Association tests revealed a smaller group of 301 species (18% of 1,666) that occurred in floodplains more often than random expectation (*P* < 0.05) and were therefore considered floodplain specialists (Supplementary Table 3). The majority of floodplain specialists had a clear preference for one type of floodplain habitat or another (*várzea*, 51%, 154 species; *igapó*, 38%, 115 species), while relatively few floodplain specialists were associated with both floodplain habitats (11%, 32 species). False positives due to multiple testing are expected for about 15 species (5% of 301 species), so we estimate the overall percentage of floodplain specialist tree species to be 17% of the 1,666 species tested. Terra firme specialists accounted for 700 species (42% of 1,666), and the remaining 665 species (40%) had no clear habitat association and were therefore considered generalists.

**Fig. 2:**
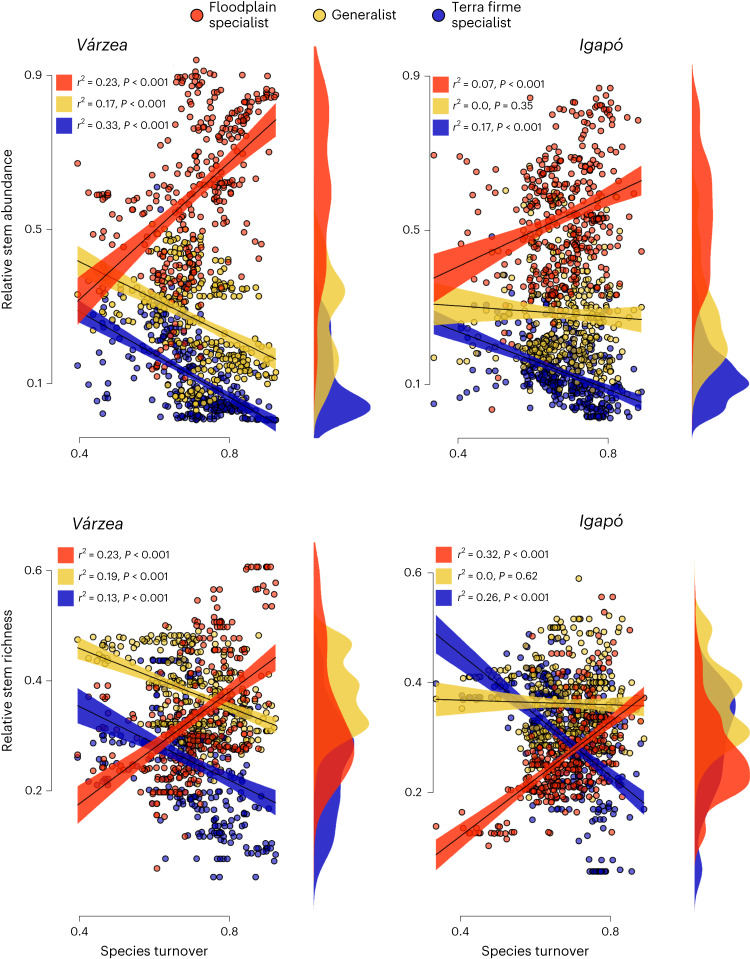
Relationships between species turnover and the relative abundance and richness of floodplain specialists, habitat generalists and spillover from terra firme (terra firme specialists) in *várzea* and *igapó*. With increasing levels of species turnover, floodplain specialists become more dominant, while spillover from terra firme species decreases. The proportions are derived from interpolated compositional grids of *várzea* and *igapó* after cross-referencing with the names of the 1,666 species tested for habitat association. The relationships with species turnover are derived from simple least squares models. The coloured boxes indicate the proportion of explained variance (*r*^2^) and *P* values. The trend lines (black) are bounded by coloured bands showing the 95% CIs. Density plots for the relative abundance and richness of each species group are shown in the right margins.

By cross-referencing the names of the 1,666 tested species with each floodplain composition grid, we were able to examine the cell-wise relative abundance and richness of floodplain specialists (301 species), habitat generalists and terra firme specialists (that is, spillover from terra firme) in floodplains. As expected, the relative abundance and richness of floodplain specialists increased with species turnover. More surprising were the upper limits of floodplain specialist rates—in areas of high species turnover, floodplain forests are decidedly specialist-dominated, with nearly half of floodplain cell richness and a majority (>70%) of floodplain stems pertaining to specialist species (Fig. 2). At low levels of species turnover, generalist species and spillover species from terra firme (terra firme specialists) together accounted for the majority of species and stems in floodplains. However, even at low levels of species turnover, floodplain specialists still account for about a third of stems and 20% of tree species richness. For any floodplain anywhere, therefore, a considerable complement of its local tree diversity is strongly circumscribed to its flooded habitat. Habitat generalists, particularly in *igapó*, can remain an important component of floodplain forests regardless of the level of species turnover. Our lower range of relative richness for habitat specialists in floodplains is remarkably similar to our previous estimate for *várzea*^7^, but our upper range (about 60%) is nearly twice as large, undoubtedly reflecting the greater amount of quantitative abundance information available in our current database.

### Optimizing conservation benefits for biodiversity

Our results suggest that although floodplains cumulatively account for only about a fifth of the regional surface area, they contain most of Amazonian tree diversity. Assuming that our sample of 1,666 species can be taken as representative of the tree flora at large, then nearly 9 of every 10 species can inhabit floodplains, albeit many of them at low densities. Importantly, a substantial fraction of Amazonian tree diversity may be strongly dependent on floodplain habitat, as our estimates indicate that nearly one in every six tree species in Amazonia is a floodplain specialist. Floodplains are a prominent feature of fine-scale habitat heterogeneity in Amazonia, which has long been a leading hypothesis for the maintenance of the region’s exceptional tree richness^3,7,8,10^. Yet, regional examinations of tree diversity have typically not taken into account the type of fine-scale habitat heterogeneity that floodplains generate^22,30^, limiting our understanding of how biodiversity is organized on landscapes. Our results perhaps illustrate more clearly than ever the substantial complement of the Amazon’s regional tree diversity that floodplains accommodate.

For most floodplain specialists identified here, detailed ecological data are still lacking. Strong habitat preference presumably reflects a high degree of adaptation that confers advantages for a particular species in a particular habitat but disadvantages in other habitats, so it is often linked to increased vulnerability to environmental change and unique ecological function. Our results indicate that together these specialists typically account for at least 20% of tree species richness or tree abundance in most floodplains, meaning that this select group of specialized species is likely to have a disproportionate influence on patterns of primary production, resource availability and forest structure that directly sustain adjacent terrestrial and aquatic biodiversity. Such findings raise questions about the safeguarding of ecological function of a critical group of several hundred floodplain specialists.

While ideas about integrating floodplains into entire watershed-based strategies have existed as general recommendations for some time, area-based conservation strategies in Amazonia have overwhelmingly focused on purely terrestrial, non-flooded habitats^14–16^. Because our maps of species turnover reflect the spatial arrangement of tree diversity on local landscapes, they offer an interesting tool that may enable us to move beyond general recommendations and towards more geographically explicit frameworks better able to place the opportunity costs of competing land-use strategies into sharper focus. For example, putting greater emphasis on capturing areas of high species turnover may better permit watershed-based conservation frameworks to identify and locate high benefit/cost opportunities for landscape-level biodiversity while optimizing benefits to future floodplain systems. According to our findings, these benefits will typically be highest along the large, lowland rivers with strong flood seasonality, where landscape-level beta diversity is high. Regrettably, on current landscapes these river attributes tend to combine high human accessibility with low protection status^31^.

### Identifying sensitive floodplains

Despite mounting evidence that basin-wide precipitation patterns and proliferating hydro-electric dams are increasingly changing key ecological aspects of river hydrology^12,13,32^, our knowledge regarding the responses of tree communities has come from a small number of sites already undergoing hydrological degradation^33^ and is thus largely retrospective and highly fragmentary. Our results may offer a viable path towards understanding the geography of floodplain sensitivity to hydrological alteration within a more anticipatory framework. For example, high species turnover implies not only high concentrations of specialized species with potentially unique ecological functioning but also narrow circumscription of species populations to floodplain habitat, putting both species and function at greater risk of local extinction, should floodplain conditions change. In areas of high species turnover, therefore, hydrological alteration will arguably be more ecologically disruptive than in other areas. We found that the spatial configuration of these vulnerable floodplains is very much concentrated in central Amazonia, a position placing them at the heart of the fluvial network. The vulnerability of these floodplains to accumulating downstream and upstream hydrological impacts that propagate from elsewhere in the basin is a concern. With up to 300 flood days annually and flood amplitudes of 9–12 m, the floristically unique forests occupying this central Amazonian region are contingent on some of the most exceptional hydrologic conditions worldwide^1^. It is doubtful that any restoration technology could reverse damaging hydrological impacts on their tree communities, the species interactions they support or the ecological services they underpin. It therefore seems clearer than ever that conserving the Amazon’s tree diversity and its ecological functioning will require both landscape-level planning that incorporates floodplain habitat into protected areas and regional strategies for water management to safeguard whole-catchment hydrological integrity.

### Limitations

We have produced a spatially explicit look at the geography of species turnover and the habitat specificity of floodplain forests that covers wide environmental and floristic variation in the Amazon basin. However, it is important to acknowledge that we have done so only at the coarsest of spatial scales. Large spatial gaps in forest inventories still exist, which we have currently resolved by interpolating species abundance information over considerable distances. Moreover, our measure of flood wave position remains a simplified estimate of more complex geographic variation in local flood regimes. Complementary approaches at smaller, basin-level spatial scales will undoubtedly reveal important local nuance not captured at our scale of analysis^18^. Our results also suggest that species turnover may be maintained in floodplains by one or more (unmeasured) factors that interact with flooding. For example, river meandering (and the resulting primary succession that follows) undoubtedly shapes the species composition of floodplain forests in important ways, but its unique contribution to maintaining species turnover with terra firme remains unknown. Similarly, the amount and fertility of sediment transported by rivers, and the allochthonous alluvial soil that develops from it, often differ considerably from those of uplands in ways that impact water and nutrient availability and therefore species composition. Ongoing hydrological degradation will considerably alter these and other unknown factors, yet their individual effects on the maintenance of species turnover remain unexplored. These limitations notwithstanding, our findings provide the most comprehensive view yet of the remarkable contribution that floodplains make to Amazonian tree diversity, offer a valuable window into the sensitivity of floodplain forests and have practical implications for floodplain conservation.

## Methods

### Environmental data

Data were obtained from publicly available gridded datasets or constructed from georeferenced data. To capture regional variability in annual flooding, we used TWS from NASA’s GRC-Tellus satellite^34^. GRC-Tellus measures spatiotemporal changes in the Earth’s gravity field, which are largely the result of alterations in water thickness near the Earth’s surface. In the Amazon region, most annual TWS variability is governed by changes in the mass of surface flood waters and thus captures the hydrological signal resulting from seasonal flooding^35^. TWS is measured in centimetres of equivalent land water thickness and is available as monthly, 1°-gridded data expressed as anomalies relative to a baseline average from January 2004 to December 2009. The data are previously corrected using an independent component analysis approach to remove north–south stripes and are post-processed with a 300-km-wide Gaussian filter (a spatial grain analogous to our community grids based on 3° interpolations) to produce smoothed solutions^26,34^. As a measure of the magnitude of seasonal flooding for each pixel, we calculated the average yearly range for TWS over a 15-year period (2002–2017).

To capture regional variation in annual rainfall, we obtained gridded data from WorldClim Bioclimatic variables (bio 12)^36^.

For analysis, environmental grids for flooding and rainfall were sampled to the same 1° grids used for the composition data, using a bilinear interpolation. For visualization in Fig. 1b, the flooding grid was resampled to higher (0.1°) resolution using a bilinear interpolation.

### Composition data

We produced three analogous compositional grids of 567 1° cells covering the Amazon region for terra firme, *várzea* and *igapó*, using tree inventory data from the ATDN^22^. ATDN inventories falling outside of the grid were excluded. The final data subset comprises 1,705 mostly 1 ha tree inventory plots with relatively complete information on species composition and abundances (Extended Data Fig. 1 and Supplementary Table 1). Only species-level identifications were retained, accounting for 85% of species and 89% of stems. Plots were classified as terra firme (*n* = 1,250, 73%), *várzea* (*n* = 271, 16%) or *igapó* (*n* = 184, 11%), following the original habitat designations of the contributors, and species stem densities in plots were standardized to stems per ha to account for variable plot areas. We used habitat-specific plot data to populate compositional grids for each habitat separately, in two alternative manners. In the first approach, species cell abundances were interpolated from abundance information in plot inventories located within a 3° search radius, using inverse distance weighting set to a power of 2 (Extended Data Fig. 2). The search radius ensures that a species is predicted to be absent in cells where it has no nearby records of occurrence, and its maximum distance set at 3° is an optimization previously determined^37^ by the match between inverse-distance-weighting maps of all species and a Fisher’s alpha-diversity map of Amazonian forests^28^. In the second approach, species cell abundances were generated by pooling plots contained inside individual grid cells, without any spatial interpolation. Whereas the approach based on spatial interpolation constructs plausible cell metacommunities using all sampling localities within ~300 km, the approach of pooling samples within individual grid cells makes considerably less use of the available data but removes spatial dependencies in the response variable.

### Species turnover

We assessed species turnover at analogous 1° cells of *várzea*–terra firme and *igapó*–terra firme grid pairs, using both interpolated and pooled versions of compositional data in parallel analyses. Species turnover is a function of the number of species shared between floodplain and terra firme samples (*a*) and the numbers of species unique to either sample (*b* and *c*). We used Simpson dissimilarity, *β*_cell_ = 1 − *a*/(*a* + min(*b*,*c*)), where min(*b*,*c*) refers to the smallest number of unique species^38^. This species turnover metric is not influenced by differences in richness that are expected between terra firme and floodplain samples and is thus more suited to our questions regarding species replacement. Species turnover at a given cell location was assessed by repeatedly subsampling analogous terra firme and floodplain cells, each time drawing 500 stems from each habitat with replacement, setting the probability of species selection proportional to cell density of each species. Although species selection is based on predicted abundance information, our presence/absence measure of species turnover is robust to highly abundant species that alternative abundance-based indices may be overly sensitive to. Each sampled assembly is therefore of similar size to a standard hectare plot and reflects average, habitat-specific tree composition on the basis of information from the nearby plot inventories. For each cell, we report the mean Simpson’s dissimilarity of 1,000 subsamples. As an additional precaution to avoid calculating species turnover at severely undersampled cells, we required cell richness for each habitat grid to be >100 species. Species tend to accumulate rapidly in tropical forests, so an accumulation of fewer than 100 species is a strong indication that sampling was either too limited or skewed towards environmentally extreme or disturbed environments. For similar reasons, for analysis based on compositional grids derived from pooling inventories, we additionally required that cells contain at least two floodplain and two terra firme inventories. In both the interpolated and pooled approaches, the total number of cells where species turnover was calculated depended on the spatial configuration of terra firme and floodplain inventories and how these overlapped geographically. Bivariate relationships of species turnover with flooding were examined using least absolute deviation (quantile regression) models. Quantile regression was preferred over least squares because initial inspection revealed flooding to be a better predictor of minimum levels of species turnover rather than mean levels. Quantile regression models of the lower bounds of species turnover (*τ* = 0.1) therefore provide more appropriate estimates of the relationship between flooding and minimum levels of species turnover, and residual levels of species turnover can be interpreted as the summed effect of unmeasured or unknown factors that interact with flooding to maintain species turnover^27,28^. To test whether annual rainfall interacted with flooding to maintain species turnover, we used least squares models, comparing competing least squares models with and without a rainfall interaction using *F* tests. Prior to running least squares regression, we checked for multi-collinearity among environmental factors by assessing variance inflation factors. Moran’s *I* was used to check for residual spatial autocorrelation. All analyses were performed in R v.4.1.2 (ref. ^39^) using custom code and the packages vegan^40^, quantreg^41^, raster^42^, rgdal^43^, gstat^44,45^ and ape^46^.

For visualization of the geographic patterns of species turnover, as well as residual levels of species turnover remaining from quantile regression models, we projected the original 1° grids onto higher-resolution (0.05°) floodplain maps using a bilinear interpolation method. To do this for *várzea* and *igapó* individually, we first delineated floodplains on the basis of ref. ^47^ and assigned floodplains to either *igapó* or *várzea* using the habitat classifications from georeferenced sampling localities in ATDN.

### Habitat association

The ecological association of species to floodplain or terra firme habitat was computed as the Pearson correlation between the vector of a species’ abundance among inventories and the binary vector of inventory habitat membership (that is, terra firme, *igapó* and *várzea*)^29^. In contrast to the alternative approach using indicator values, Pearson correlation additionally takes into account absences outside the target habitat and is arguably preferable for determining the ecological preference of a given species among a set of alternative habitats^29,48^. Because some species may associate with more than one floodplain habitat, we allowed for floodplain combinations (for example, *igapó* + *várzea*). A permutation test was used to evaluate statistical significance for the habitat or combination for which the correlation was highest, implemented with the multipatt function of the R package indicspecies, using the r.g option^29,48^. Differences in sampling effort among habitats, which can influence estimated coefficients, were accounted for using a group‐based stratified resampling procedure^48^. Most species have smaller ranges than the extent of the study area, so species permutations were performed on the subset of plot inventories contained within cells predicting >0 abundance, on the basis of 3° inverse-distance-weighted interpolations of plot abundance information. Only species occurring in over 20 inventories were assessed to ensure sufficient sampling. We used a *P* < 0.05 threshold to identify habitat specialists; all others were considered as habitat generalists.

### Reporting summary

Further information on research design is available in the Nature Portfolio Reporting Summary linked to this article.

### Supplementary information

Supplementary Information Supplementary Discussion 1, Fig. 1 and Table 2.

Reporting Summary

Supplementary Tables Supplementary Tables 1 and 3.

## Data Availability

Metadata for all plots used in this study are available in Supplementary Table 1. Habitat correlation scores for 1,666 species are available in Supplementary Table 3. GRC-Tellus data on monthly land water thickness are publicly available from https://grace.jpl.nasa.gov/data/get-data/monthly-mass-grids-land/. WorldClim Bioclimatic data are available from https://www.worldclim.org/data/bioclim.html. All tree inventory data can also be made available upon reasonable request to H.t.S.

## Code availability

The R scripts used in the analysis are available upon reasonable request to J. E. Householder.
