# Author Correction: One sixth of Amazonian tree diversity is dependent on river floodplains

**DOI:** 10.1038/s41559-024-02400-0

**Published:** 2024-04-02

**Authors:** John Ethan Householder, Florian Wittmann, Jochen Schöngart, Maria Teresa Fernandez Piedade, Wolfgang J. Junk, Edgardo Manuel Latrubesse, Adriano Costa Quaresma, Layon O. Demarchi, Guilherme de S. Lobo, Daniel P. P. de Aguiar, Rafael L. Assis, Aline Lopes, Pia Parolin, Iêda Leão do Amaral, Luiz de Souza Coelho, Francisca Dionízia de Almeida Matos, Diógenes de Andrade Lima Filho, Rafael P. Salomão, Carolina V. Castilho, Juan Ernesto Guevara-Andino, Marcelo de Jesus Veiga Carim, Oliver L. Phillips, Dairon Cárdenas López, William E. Magnusson, Daniel Sabatier, Juan David Cardenas Revilla, Jean-François Molino, Mariana Victória Irume, Maria Pires Martins, José Renan da Silva Guimarães, José Ferreira Ramos, Domingos de Jesus Rodrigues, Olaf S. Bánki, Carlos A. Peres, Nigel C. A. Pitman, Joseph E. Hawes, Everton José Almeida, Luciane Ferreira Barbosa, Larissa Cavalheiro, Márcia Cléia Vilela dos Santos, Bruno Garcia Luize, Evlyn Márcia Moraes de Leão Novo, Percy Núñez Vargas, Thiago Sanna Freire Silva, Eduardo Martins Venticinque, Angelo Gilberto Manzatto, Neidiane Farias Costa Reis, John Terborgh, Katia Regina Casula, Flávia R. C. Costa, Euridice N. Honorio Coronado, Abel Monteagudo Mendoza, Juan Carlos Montero, Ted R. Feldpausch, Gerardo A. Aymard C, Chris Baraloto, Nicolás Castaño Arboleda, Julien Engel, Pascal Petronelli, Charles Eugene Zartman, Timothy J. Killeen, Lorena Maniguaje Rincón, Beatriz S. Marimon, Ben Hur Marimon-Junior, Juliana Schietti, Thaiane R. Sousa, Rodolfo Vasquez, Bonifacio Mostacedo, Dário Dantas do Amaral, Hernán Castellanos, Marcelo Brilhante de Medeiros, Marcelo Fragomeni Simon, Ana Andrade, José Luís Camargo, William F. Laurance, Susan G. W. Laurance, Emanuelle de Sousa Farias, Maria Aparecida Lopes, José Leonardo Lima Magalhães, Henrique Eduardo Mendonça Nascimento, Helder Lima de Queiroz, Roel Brienen, Pablo R. Stevenson, Alejandro Araujo-Murakami, Tim R. Baker, Bruno Barçante Ladvocat Cintra, Yuri Oliveira Feitosa, Hugo F. Mogollón, Janaína Costa Noronha, Flávia Rodrigues Barbosa, Rainiellen de Sá Carpanedo, Joost F. Duivenvoorden, Miles R. Silman, Leandro Valle Ferreira, Carolina Levis, José Rafael Lozada, James A. Comiskey, Freddie C. Draper, José Julio de Toledo, Gabriel Damasco, Nállarett Dávila, Roosevelt García-Villacorta, Alberto Vicentini, Fernando Cornejo Valverde, Alfonso Alonso, Luzmila Arroyo, Francisco Dallmeier, Vitor H. F. Gomes, Eliana M. Jimenez, David Neill, Maria Cristina Peñuela Mora, Fernanda Antunes Carvalho, Fernanda Coelho de Souza, Kenneth J. Feeley, Rogerio Gribel, Marcelo Petratti Pansonato, Marcos Ríos Paredes, Jos Barlow, Erika Berenguer, Kyle G. Dexter, Joice Ferreira, Paul V. A. Fine, Marcelino Carneiro Guedes, Isau Huamantupa-Chuquimaco, Juan Carlos Licona, Toby Pennington, Boris Eduardo Villa Zegarra, Vincent Antoine Vos, Carlos Cerón, Émile Fonty, Terry W. Henkel, Paul Maas, Edwin Pos, Marcos Silveira, Juliana Stropp, Raquel Thomas, Doug Daly, William Milliken, Guido Pardo Molina, Ima Célia Guimarães Vieira, Bianca Weiss Albuquerque, Wegliane Campelo, Thaise Emilio, Alfredo Fuentes, Bente Klitgaard, José Luis Marcelo Pena, Priscila F. Souza, J. Sebastián Tello, Corine Vriesendorp, Jerome Chave, Anthony Di Fiore, Renato Richard Hilário, Luciana de Oliveira Pereira, Juan Fernando Phillips, Gonzalo Rivas-Torres, Tinde R. van Andel, Patricio von Hildebrand, William Balee, Edelcilio Marques Barbosa, Luiz Carlos de Matos Bonates, Hilda Paulette Dávila Doza, Ricardo Zárate Gómez, Therany Gonzales, George Pepe Gallardo Gonzales, Bruce Hoffman, André Braga Junqueira, Yadvinder Malhi, Ires Paula de Andrade Miranda, Linder Felipe Mozombite-Pinto, Adriana Prieto, Agustín Rudas, Ademir R. Ruschel, Natalino Silva, César I. A. Vela, Stanford Zent, Egleé L. Zent, Angela Cano, Yrma Andreina Carrero Márquez, Diego F. Correa, Janaina Barbosa Pedrosa Costa, Bernardo Monteiro Flores, David Galbraith, Milena Holmgren, Michelle Kalamandeen, Marcelo Trindade Nascimento, Alexandre A. Oliveira, Hirma Ramirez-Angulo, Maira Rocha, Veridiana Vizoni Scudeller, Rodrigo Sierra, Milton Tirado, Maria Natalia Umaña, Geertje van der Heijden, Emilio Vilanova Torre, Manuel Augusto Ahuite Reategui, Cláudia Baider, Henrik Balslev, Sasha Cárdenas, Luisa Fernanda Casas, William Farfan-Rios, Cid Ferreira, Reynaldo Linares-Palomino, Casimiro Mendoza, Italo Mesones, Germaine Alexander Parada, Armando Torres-Lezama, Ligia Estela Urrego Giraldo, Daniel Villarroel, Roderick Zagt, Miguel N. Alexiades, Edmar Almeida de Oliveira, Karina Garcia-Cabrera, Lionel Hernandez, Walter Palacios Cuenca, Susamar Pansini, Daniela Pauletto, Freddy Ramirez Arevalo, Adeilza Felipe Sampaio, Elvis H. Valderrama Sandoval, Luis Valenzuela Gamarra, Hans ter Steege

**Affiliations:** 1 Wetland Department, Institute of Geography and Geoecology, Karlsruhe Institute of Technology, Rastatt, Germany; 2 Ecology, Monitoring and Sustainable Use of Wetlands, Instituto Nacional de Pesquisas da Amazônia, Manaus, Brazil; 3 National Institute for Science and Technology of Wetlands, Federal University of Mato Grosso, Cuiabá, Brazil; 4 Environmental Sciences Graduate Program-CIAMB, Federal University of Goiás, Goiânia, Brazil; 5 Procuradoria-Geral de Justiça, Ministério Público do Estado do Amazonas, Manaus, Brazil; 6 Coordenação de Dinâmica Ambiental, Instituto Nacional de Pesquisas da Amazônia, Manaus, Brazil; 7 Biodiversity and Ecosystem Services, Instituto Tecnológico Vale, Belém, Brazil; 8 Department of Ecology, Institute of Biological Sciences, University of Brasilia, Brasilia, Brazil; 9 Biocentre Klein Flottbek and Botanical Gardens, University of Hamburg, Hamburg, Germany; 10 Coordenação de Biodiversidade, Instituto Nacional de Pesquisas da Amazônia, Manaus, Brazil; 11 Programa Professor Visitante Nacional Sênior na Amazônia—CAPES, Universidade Federal Rural da Amazônia, Belém, Brazil; 12 Coordenação de Botânica, Museu Paraense Emílio Goeldi, Belém, Brazil; 13 Centro de Pesquisa Agroflorestal de Roraima, Embrapa Roraima, Boa Vista, Brazil; 14 Grupo de Investigación en Ecología y Evolución en los Trópicos, Universidad de las Américas, Quito, Ecuador; 15 Keller Science Action Center, the Field Museum, Chicago, IL USA; 16 Departamento de Botânica, Instituto de Pesquisas Científicas e Tecnológicas do Amapá, Macapá, Brazil; 17 School of Geography, University of Leeds, Leeds, UK; 18 Herbario Amazónico Colombiano, Instituto SINCHI, Bogotá, Colombia; 19 Coordenação de Pesquisas em Ecologia, Instituto Nacional de Pesquisas da Amazônia, Manaus, Brazil; 20 AMAP, IRD, Cirad, CNRS, INRAE, Université de Montpellier, Montpellier, France; 21 Amcel Amapá Florestal e Celulose S.A, Santana, Brazil; 22 ICNHS, Federal University of Mato Grosso, Sinop, Brazil; 23 Catalogue of Life, Leiden, the Netherlands; 24 School of Environmental Sciences, University of East Anglia, Norwich, UK; 25 Science and Education, the Field Museum, Chicago, IL USA; 26 Institute of Science and Environment, University of Cumbria, Ambleside, UK; 27 ICNHS, Universidade Federal de Mato Grosso, Sinop, Brazil; 28 Departamento de Biologia Vegetal, Instituto de Biologia, Universidade Estadual de Campinas, Campinas, Brazil; 29 Divisao de Sensoriamento Remoto, Instituto Nacional de Pesquisas Espaciais, São José dos Campos, Brazil; 30 Herbario Vargas, Universidad Nacional de San Antonio Abad del Cusco, Cusco, Peru; 31 Biological and Environmental Sciences, University of Stirling, Stirling, UK; 32 Centro de Biociências, Departamento de Ecologia, Universidade Federal do Rio Grande do Norte, Natal, Brazil; 33 Departamento de Biologia, Universidade Federal de Rondônia, Porto Velho, Brazil; 34 Programa de Pós-Graduação em Biodiversidade e Biotecnologia PPG- Bionorte, Universidade Federal de Rondônia, Porto Velho, Brazil; 35 Department of Biology and Florida Museum of Natural History, University of Florida, Gainesville, FL USA; 36 Centre for Tropical Environmental and Sustainability Science and College of Science and Engineering, James Cook University, Cairns, Queensland Australia; 37 Instituto de Investigaciones de la Amazonía Peruana, Iquitos, Peru; 38 School of Geography and Sustainable Development, University of St Andrews, St Andrews, UK; 39 Jardín Botánico de Missouri, Oxapampa, Peru; 40 Instituto Boliviano de Investigacion Forestal, Santa Cruz, Bolivia; 41 Geography, College of Life and Environmental Sciences, University of Exeter, Exeter, UK; 42 Programa de Ciencias del Agro y el Mar, Herbario Universitario (PORT), UNELLEZ-Guanare, Guanare, Venezuela; 43 International Center for Tropical Botany Department of Biological Sciences, Florida International University, Miami, FL USA; 44 Cirad UMR Ecofog, AgrosParisTech, CNRS, INRAE, Université de Guyane, Kourou, France; 45 Agteca-Amazonica, Santa Cruz, Bolivia; 46 Programa de Pós-Graduação em Ecologia e Conservação, Universidade do Estado de Mato Grosso, Nova Xavantina, Brazil; 47 Programa de Pós-Graduação em Ecologia, Instituto Nacional de Pesquisas da Amazônia, Manaus, Brazil; 48 Facultad de Ciencias Agrícolas, Universidad Autónoma Gabriel René Moreno, Santa Cruz, Bolivia; 49 Centro de Investigaciones Ecológicas de Guayana, Universidad Nacional Experimental de Guayana, Bolivar, Venezuela; 50 Embrapa Recursos Genéticos e Biotecnologia, Parque Estação Biológica, Prédio da Botânica e Ecologia, Brasilia, Brazil; 51 Projeto Dinâmica Biológica de Fragmentos Florestais, Instituto Nacional de Pesquisas da Amazônia, Manaus, Brazil; 52 Laboratório de Ecologia de Doenças Transmissíveis da Amazônia, Instituto Leônidas e Maria Deane, Fiocruz, Manaus, Brazil; 53 Programa de Pós-graduação em Biodiversidade e Saúde, Instituto Oswaldo Cruz, Rio de Janeiro, Brazil; 54 Instituto de Ciências Biológicas, Universidade Federal do Pará, Belém, Brazil; 55 Programa de Pós-Graduação em Ecologia, Universidade Federal do Pará, Belém, Brazil; 56 Empresa Brasileira de Pesquisa Agropecuária, Embrapa Amazônia Oriental, Belém, Brazil; 57 Diretoria Técnico-Científica, Instituto de Desenvolvimento Sustentável Mamirauá, Tefé, Brazil; 58 Laboratorio de Ecología de Bosques Tropicales y Primatología, Universidad de los Andes, Bogotá, Colombia; 59 Museo de Historia Natural Noel Kempff Mercado, Universidad Autónoma Gabriel Rene Moreno, Santa Cruz, Bolivia; 60 Birmingham Institute for Forest Research, University of Birmingham, Birmingham, UK; 61 Programa de Pós-Graduação em Biologia (Botânica), Instituto Nacional de Pesquisas da Amazônia, Manaus, Brazil; 62 Endangered Species Coalition, Silver Spring, MD USA; 63 Institute of Biodiversity and Ecosystem Dynamics, University of Amsterdam, Amsterdam, the Netherlands; 64 Biology Department and Center for Energy, Environment and Sustainability, Wake Forest University, Winston Salem, NC USA; 65 Graduate Program in Ecology, Federal University of Santa Catarina, Florianópolis, Brazil; 66 Facultad de Ciencias Forestales y Ambientales, Instituto de Investigaciones para el Desarrollo Forestal, Universidad de los Andes, Mérida, Venezuela; 67 Inventory and Monitoring Program, National Park Service, Fredericksburg, VA USA; 68 Center for Conservation and Sustainability, Smithsonian Conservation Biology Institute, Washington, DC USA; 69 Department of Geography and Planning, University of Liverpool, Liverpool, UK; 70 Ciências Ambientais, Universidade Federal do Amapá, Macapá, Brazil; 71 Gothenburg Global Biodiversity Centre, University of Gothenburg, Gothenburg, Sweden; 72 Programa Restauración de Ecosistemas, Centro de Innovación Científica Amazónica, Tambopata, Peru; 73 Peruvian Center for Biodiversity and Conservation, Iquitos, Peru; 74 Andes to Amazon Biodiversity Program, Madre de Dios, Peru; 75 Escola de Negócios Tecnologia e Inovação, Centro Universitário do Pará, Belém, Brazil; 76 Environmental Science Program, Geosciences Department, Universidade Federal do Pará, Belém, Brazil; 77 Grupo de Ecología y Conservación de Fauna y Flora Silvestre, Instituto Amazónico de Investigaciones Imani, Universidad Nacional de Colombia sede Amazonia, Leticia, Colombia; 78 Universidad Estatal Amazónica, Puyo, Ecuador; 79 Universidad Regional Amazónica IKIAM, Tena, Ecuador; 80 Instituto de Ciências Biológicas, Departamento de Genética, Ecologia e Evolução, Universidade Federal de Minas Gerais, Belo Horizonte, Brazil; 81 Department of Biology, University of Miami, Coral Gables, FL USA; 82 Fairchild Tropical Botanic Garden, Coral Gables, FL USA; 83 Instituto de Biociências, Departamento de Ecologia, Universidade de Sao Paulo, São Paulo, Brazil; 84 Servicios de Biodiversidad EIRL, Iquitos, Peru; 85 Lancaster Environment Centre, Lancaster University, Lancaster, UK; 86 Environmental Change Institute, University of Oxford, Oxford, UK; 87 School of Geosciences, University of Edinburgh, Edinburgh, UK; 88 Tropical Diversity Section, Royal Botanic Garden Edinburgh, Edinburgh, UK; 89 Department of Integrative Biology, University of California, Berkeley, Berkeley, CA USA; 90 Empresa Brasileira de Pesquisa Agropecuária, Embrapa Amapá, Macapá, Brazil; 91 Herbario HAG, Universidad Nacional Amazónica de Madre de Dios, Puerto Maldonado, Peru; 92 Direccíon de Evaluación Forestal y de Fauna Silvestre, Magdalena del Mar, Peru; 93 Instituto de Investigaciones Forestales de la Amazonía, Universidad Autónoma del Beni José Ballivián, Riberalta, Bolivia; 94 Escuela de Biología Herbario Alfredo Paredes, Universidad Central, Quito, Ecuador; 95 Office national des forêts, Direction régionale de la Guyane, Cayenne, French Guiana; 96 Department of Biological Sciences, California State Polytechnic University, Arcata, CA USA; 97 Naturalis Biodiversity Center, Leiden, the Netherlands; 98 Quantitative Biodiversity Dynamics, Utrecht University, Utrecht, the Netherlands; 99 Utrecht University Botanic Gardens, Utrecht, the Netherlands; 100 Centro de Ciências Biológicas e da Natureza, Universidade Federal do Acre, Rio Branco, Brazil; 101 Biogeography Department, Trier University, Trier, Germany; 102 Iwokrama International Centre for Rain Forest Conservation and Development, Georgetown, Guyana; 103 New York Botanical Garden, Bronx, New York, NY USA; 104 Department for Ecosystem Stewardship, Royal Botanic Gardens, Kew, Richmond, UK; 105 Herbario Nacional de Bolivia, Universitario UMSA, La Paz, Bolivia; 106 Center for Conservation and Sustainable Development, Missouri Botanical Garden, St. Louis, MO USA; 107 Department for Accelerated Taxonomy, Royal Botanic Gardens, Kew, Richmond, UK; 108 Universidad Nacional de Jaén, Cajamarca, Peru; 109 Laboratoire Evolution et Diversité Biologique, CNRS and Université Paul Sabatier, UMR 5174 EDB, Toulouse, France; 110 Department of Anthropology, University of Texas at Austin, Austin, TX USA; 111 Estación de Biodiversidad Tiputini, Colegio de Ciencias Biológicas y Ambientales, Universidad San Francisco de Quito, Quito, Ecuador; 112 Fundación Puerto Rastrojo, Bogotá, Colombia; 113 Department of Wildlife Ecology and Conservation, University of Florida, Gainesville, FL USA; 114 Biosystematics Group, Wageningen University, Wageningen, the Netherlands; 115 Fundación Estación de Biología, Bogotá, Colombia; 116 Department of Anthropology, Tulane University, New Orleans, LA USA; 117 PROTERRA, Instituto de Investigaciones de la Amazonía Peruana, Iquitos, Peru; 118 ACEER Foundation, Puerto Maldonado, Peru; 119 Amazon Conservation Team, Arlington, VA USA; 120 Institut de Ciència i Tecnologia Ambientals, Universitat Autònoma de Barcelona, Barcelona, Spain; 121 Environmental Change Institute, Oxford University Centre for the Environment, Oxford, UK; 122 Instituto de Ciencias Naturales, Universidad Nacional de Colombia, Bogotá, Colombia; 123 Instituto de Ciência Agrárias, Universidade Federal Rural da Amazônia, Belém, Brazil; 124 Escuela Profesional de Ingeniería Forestal, Universidad Nacional de San Antonio Abad del Cusco, Puerto Maldonado, Peru; 125 Laboratory of Human Ecology, Instituto Venezolano de Investigaciones Científicas, Caracas, Venezuela; 126 Cambridge University Botanic Garden, Cambridge University, Cambridge, UK; 127 Programa de Maestria de Manejo de Bosques, Universidad de los Andes, Mérida, Venezuela; 128 Centre for Biodiversity and Conservation Science, University of Queensland, Brisbane, Queensland Australia; 129 Resource Ecology Group, Wageningen University & Research, Wageningen, the Netherlands; 130 School of Earth, Environment and Society, McMaster University, Hamilton, Ontario Canada; 131 Laboratório de Ciências Ambientais, Universidade Estadual do Norte Fluminense, Campos dos Goytacazes, Brazil; 132 Instituto de Investigaciones para el Desarrollo Forestal, Universidad de los Andes, Mérida, Venezuela; 133 Departamento de Biologia, Instituto de Ciências Biológicas, Universidade Federal do Amazonas, Manaus, Brazil; 134 GeoIS, Quito, Ecuador; 135 Department of Ecology and Evolutionary Biology, University of Michigan, Ann Arbor, MI USA; 136 Faculty of Social Sciences, University of Nottingham, Nottingham, UK; 137 Wildlife Conservation Society, New York, NY USA; 138 Medio Ambiente, PLUSPRETOL, Iquitos, Peru; 139 Mauritius Herbarium, Agricultural Services, Ministry of Agro-Industry and Food Security, Moka, Mauritius; 140 Department of Biology, Aarhus University, Aarhus, Denmark; 141 Escuela de Ciencias Forestales, Universidad Mayor de San Simon, Cochabamba, Bolivia; 142 FOMABO, Manejo Forestal en las Tierras Tropicales de Bolivia, Cochabamba, Bolivia; 143 Departamento de Ciencias Forestales, Universidad Nacional de Colombia, Medellín, Colombia; 144 Fundación Amigos de la Naturaleza, Santa Cruz, Bolivia; 145 Tropenbos International, Ede, the Netherlands; 146 School of Anthropology and Conservation, University of Kent, Canterbury, UK; 147 Herbario Nacional del Ecuador, Universidad Técnica del Norte, Quito, Ecuador; 148 Instituto de Biodiversidade e Florestas, Universidade Federal do Oeste do Pará, Santarém, Brazil; 149 Facultad de Biologia, Universidad Nacional de la Amazonia Peruana, Iquitos, Peru; 150 Department of Biology, University of Missouri, St. Louis, MO USA

**Keywords:** Biodiversity, Community ecology

Correction to: *Nature Ecology & Evolution* 10.1038/s41559-024-02364-1, published online 11 March 2024.

In the version of the article initially published, the affiliation of Edgardo Manuel Latrubesse was incorrect and has now been amended to Environmental Sciences Graduate Program-CIAMB, Federal University of Goiás, Goiânia, Brazil in the HTML and PDF versions of the article.

