# Supplementary material for: One sixth of Amazonian tree diversity is dependent on river floodplains
Source: Nat Ecol Evol. 2024 Mar 11;8(5):901–11. doi: 10.1038/s41559-024-02364-1 (PMC11090827; doi:10.1038/s41559-024-02364-1)
Supplement: Supplementary file 1 — Supplementary Discussion 1, Fig. 1 and Table 2. [file 41559_2024_2364_MOESM1_ESM.pdf]

nature ecology & evolution

Article

https://doi.org/10.1038/s41559-024-02364-1

One sixth of Amazonian tree diversity is dependent on river floodplains

In the format provided by the authors and unedited

Supplementary Discussion 1. While broad-scale patterns of species turnover corresponded to floodplain position along the flood wave, considerable variation remained unexplained. Geographically, much of this residual variation was concentrated in *várzeas* of western Amazonia, which maintained relatively high species turnover despite a low position on the flood wave. Rivers in this area drain vast catchments that are exposed to some of the highest annual precipitation rates in Amazonia, and we suspected that this might contribute to local inundation patterns in ways that maintain elevated species turnover (Supplementary Figure 1a & b). For example, greater rainfall might keep floodplain forests on typically clayey, poorly-drained alluvium waterlogged for a longer period than surrounding and better-drained terra firme, or, alternatively, influence river flooding in ways that support higher species turnover. To investigate this, we incorporated both flooding and annual rainfall in least squares models, using F tests to ask whether they explained more variation in species turnover than models with flooding alone. The effect of rainfall should be interactive, with a larger influence on species turnover at lower positions of the flood wave. It is here where flooding is less limiting to the degree of compositional overlap between floodplains and terra firme, and where much of the unexplained variation remains. At high positions of the flood wave, greater flooding always limits compositional overlap and leads to relatively high species turnover.

We found some statistical support for this. Both *várzea* and *igapó*, interaction models performed better than those with flooding alone, principally because regions positioned low on the flood wave tended to support higher species turnover when they were rainier, than when they were drier (Supplementary Figure 1c & d). At high positions on the flood wave, species turnover is constrained to relatively high levels in both ecosystems, although for *várzea*, rainier regions were predicted to have slightly lower species turnover than drier regions. The amount of explained variation was still modest (*várzea;* $F = 58.87$, $r^2 = 0.37$, $df = 3, 298$, $p < 0.001$,; *igapó;* $F = 17.1$,

$r^2 = 0.13$, df = 3, 346, $p < 0.001$) and some residual spatial autocorrelation remained in both interaction models. We therefore reassessed relationships using species turnover derived from pooled inventories, finding that although interaction models were marginally significant, F tests supported more parsimonious models with flooding alone, for both *várzea* ($F = 5.2$, $r^2 = 0.19$, df = 23, $p < 0.05$) and *igapó* ($F = 6.4$, $r^2 = 0.24$, df = 20, $p < 0.05$) (Supplementary Table 2). The stricter sampling protocol therefore reconfirms the key influence of flooding, but because it results in barely two dozen cells where terra firme and floodplain forests can be compared, patterns of species turnover across interacting environmental gradients remain difficult to determine with greater certainty.

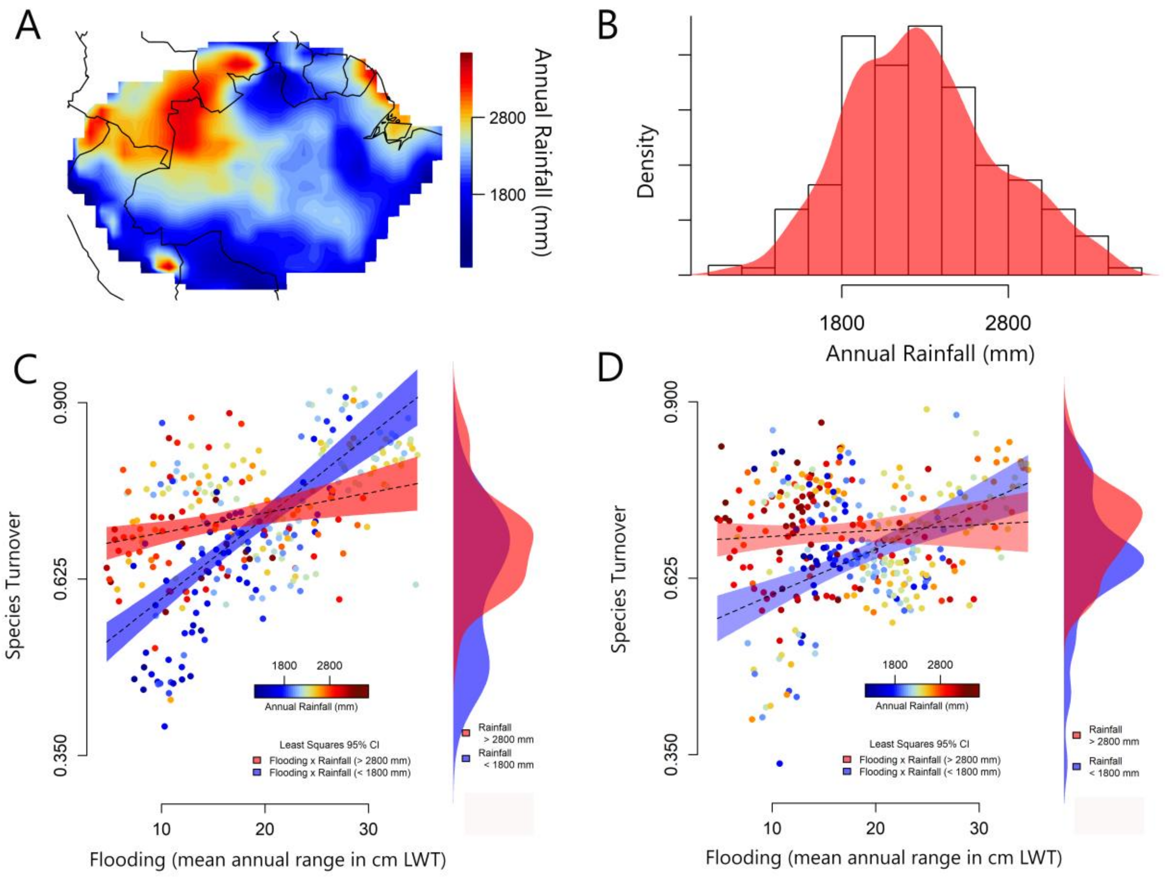

Supplementary Figure 1. Interactive effects between flooding and annual rainfall suggest that areas with high annual rainfall tend to maintain elevated levels of species turnover despite a low

position on the flood wave. (**A**) Spatial variation and (**B**) distribution of annual rainfall in Amazonia. The distribution of the level species turnover between terra firme and (**C**) *várzea*, or (**D**) *igapó* along the flood wave. Model predictions and 95% confidence interval bands are derived from a least squares regression with flooding and annual precipitation as interacting variables. Prediction lines in red correspond to areas with high annual rainfall, defined as the 80th percentile of annual rainfall (2,800 mm). Predictions in blue correspond areas with low annual rainfall, defined as the 20th percentile (1,800 mm). Marginal histograms of species turnover show the distribution of species turnover levels for the subset of grid cells above 2,800 mm (red) and below 1,800 mm (blue) rainfall thresholds.

			Least Squares Results			Model Comparisons with F tests				
Compositional Grid	Species Turnover	Model	r^2	p value	Moran I	RSS	Res DF	F	df	p value
Interpolated	V-TF	F	0.28	<0.001	0.15*	3.79	300	-	-	-
		**F x R**	0.37	<0.001	0.09*	3.28	298	23.2	2	<0.001
	I-TF	F	0.09	<0.001	0.13*	4.11	348	-	-	-
		**F x R**	0.13	<0.001	0.12*	3.94	346	7.7	2	<0.001
Pooled	V-TF	**F**	0.19	0.03	0.005	0.47	23	-	-	-
		F x R	0.28	0.06	-0.06	0.41	21	1.5	2	0.24
	I-TF	**F**	0.24	0.02	-0.01	0.27	20	-	-	-
		F x R	0.35	0.04	-0.03	0.23	18	1.5	2	0.23

Supplementary Table 2. Comparison of competing ordinary least squares environmental models of species turnover. Using F tests, simple flooding (F) models were compared against more complex models that included an interaction with annual rainfall (F x R). Four grids of species turnover were assessed, which included both interpolated and pooled versions of *várzea*-terra firme (V-TF) and *igapó*-terra firme (I-TF) grids. From each grid of species turnover, selected environmental models are in bold, showing that rainfall interactions are supported for interpolated compositional data, but not pooled data. For each model, the explained fraction of variation (r^2), p value, and Moran's I are also shown. Moran's I indicates residual spatial autocorrelation (* = $p < 0.001$).
