# Supplementary material for: One sixth of Amazonian tree diversity is dependent on river floodplains
Source: Nat Ecol Evol. 2024 Mar 11;8(5):901–11. doi: 10.1038/s41559-024-02364-1 (PMC11090827; doi:10.1038/s41559-024-02364-1)
Supplement: Supplementary file 2 — Reporting Summary [file 41559_2024_2364_MOESM2_ESM.pdf]

nature portfolio

Corresponding author(s): John Ethan Householder

Last updated by author(s): Mar 27, 2023

Reporting Summary

Nature Portfolio wishes to improve the reproducibility of the work that we publish. This form provides structure for consistency and transparency in reporting. For further information on Nature Portfolio policies, see our Editorial Policies and the Editorial Policy Checklist.

Statistics

For all statistical analyses, confirm that the following items are present in the figure legend, table legend, main text, or Methods section.

n/a	Confirmed	
☐	☒	The exact sample size (*n*) for each experimental group/condition, given as a discrete number and unit of measurement
☐	☒	A statement on whether measurements were taken from distinct samples or whether the same sample was measured repeatedly
☐	☒	The statistical test(s) used AND whether they are one- or two-sided<br>*Only common tests should be described solely by name; describe more complex techniques in the Methods section.*
☐	☒	A description of all covariates tested
☐	☒	A description of any assumptions or corrections, such as tests of normality and adjustment for multiple comparisons
☐	☒	A full description of the statistical parameters including central tendency (e.g. means) or other basic estimates (e.g. regression coefficient) AND variation (e.g. standard deviation) or associated estimates of uncertainty (e.g. confidence intervals)
☐	☒	For null hypothesis testing, the test statistic (e.g. *F*, *t*, *r*) with confidence intervals, effect sizes, degrees of freedom and *P* value noted<br>*Give P values as exact values whenever suitable.*
☒	☐	For Bayesian analysis, information on the choice of priors and Markov chain Monte Carlo settings
☒	☐	For hierarchical and complex designs, identification of the appropriate level for tests and full reporting of outcomes
☐	☒	Estimates of effect sizes (e.g. Cohen's *d*, Pearson's *r*), indicating how they were calculated

Our web collection on statistics for biologists contains articles on many of the points above.

Software and code

Policy information about availability of computer code

Data collection

No specific software was used to collect the data.
Vegetation data was collected in the field.
Environmental data was collected from publically available sources.

Data analysis

All analyese were perfomed in R -- R Core Team, R: A language and environment for statistical computing. R Foundation for Statistical Computing, Vienna, Austria (2021). All standard statisical tests (ordinary least squares regressions, F-tests) were done using base R tools.

We also used a number of standard R packages for data manipulation, analysis, visualization, and statistical analysis --

For basic datatable wrangling of plot inventory data --
vegan package -- Oksanen, J. et al. vegan: Community ecology package. R package version 2.5-7. (2020).

For quantile regression --
quantreg package -- Koenker, R. quantreg: Quantile regression. R package version 5.88 (2022).

For mapping visualization and geospatial data manipulation --
raster package -- Hijmans, R.J. raster: Geographic data analysis and modeling (2021).
rgdal package -- Bivand, R., Keitt, T. & Rowlingson, B. rgdal: Bindings for the 'Geospatial' data abstraction library. (2021).

For spatial interpolation of species abundances --
gstat package -- Pebesma, E.J. Multivariable geostatistics in S: the gstat package. Computers and Geosciences 30, 683-691 (2004).

For basic tools required by other packages --
ape package -- Paradis, E. & Schliep, K. ape 5.0: an environment for modern phylogenetics and evolutionary analyses in R. Bioinformatics 35, 526-528 (2019).

For the indicator species analysis (package indicspecies)
indicspecies package -- De Cáceres, M. & Legendre, P. Associations between species and groups of sites: indices and statistical inference. Ecology 90, 3566-3574 (2009).

For manuscripts utilizing custom algorithms or software that are central to the research but not yet described in published literature, software must be made available to editors and reviewers. We strongly encourage code deposition in a community repository (e.g. GitHub). See the Nature Portfolio guidelines for submitting code & software for further information.

Data

Policy information about availability of data

All manuscripts must include a data availability statement. This statement should provide the following information, where applicable:

- Accession codes, unique identifiers, or web links for publicly available datasets
- A description of any restrictions on data availability
- For clinical datasets or third party data, please ensure that the statement adheres to our policy

The tree inventory data and code are available upon reasonable request.

Human research participants

Policy information about studies involving human research participants and Sex and Gender in Research.

Reporting on sex and gender	There are no human participants.
Population characteristics	There are no human participants.
Recruitment	There are no human participants.
Ethics oversight	There are no human participants.

Note that full information on the approval of the study protocol must also be provided in the manuscript.

Field-specific reporting

Please select the one below that is the best fit for your research. If you are not sure, read the appropriate sections before making your selection.

☐ Life sciences ☐ Behavioural & social sciences ☒ Ecological, evolutionary & environmental sciences

For a reference copy of the document with all sections, see nature.com/documents/nr-reporting-summary-flat.pdf

Ecological, evolutionary & environmental sciences study design

All studies must disclose on these points even when the disclosure is negative.

Study description	We examine the geographic patterns and environmental drivers of beta diversity between seasonal floodplain forests and adjacent terra firme forests in the Amazon region.
Research sample	We used tree inventory data from the Amazon Tree Diversity Network (ATDN), which collates tree inventories from hundreds of collaborators. The research is based on 1,715 mostly 1-ha inventories in both floodplain and terra firme habitats. Inventory information includes species names and abundances. This underlying data was sampled in a variety of ways (see below).
Sampling strategy	We had three sampling stategies<br>1) Inventory data on species abundances of floodplain and terra firme habitats were projected (using inverse distance weighting) onto separate 1-degree grids spanning the Amazon, so that each floodplain or terra frime grid cell contains information on species composition and abundance of species, based on nearby inventories. Resulting floodplain and terra firme grids were overlaid and beta diversity calculated at each cell, producing a gridded betadiversity map. Grid cells of this beta diversity product were the basic sampling unit, and sampling size was determined by the geographic distirbution of floodplains, terra firme, and their overlap.<br>2) Same as 1, but no initital projection of species abundance information was performed. This second sampling strategy was performed to limit potential issues with spatially autocorrelation of model residuals. Species abundance information cell for floodplains and terra firme was pooled inside each grid cell, and beta diversity calculated at each grid cell that included both floodplain and terra firme information of species abundance. Grid cells of this beta diversity product were the basic sampling unit, and sampling size was determined by the geographic distirbution of floodplains, terra firme, and their overlap.

	3) Species indicator analyses were performed for each species separately. This is a plot-based approach, where all inventories within a species range were utilized. Sample units are therefore inventories, a pvalues determined by randomization of habitat designations.
Data collection	Vegetation data was collected in the field, over a number of years, and by hundreds of collaborators (co-authors).
Timing and spatial scale	Only inventories in the Amazon region were included.
Data exclusions	Inventories outsize the Amazon region were excluded, as inventories outside this region have very different environmental conditions and could bias the results. This exlcusion criteria was pre-established. Individuals without species-level identifications were also excluded, representing about 10% of individuals. We cannot calculate betadiversity without species names. This exclusion rate is typical for Amazonia.
Reproducibility	In the manuscript we employ analytical strategies with important differences in methodological choices, so the question of reproducibility is reported in the manuscript. We report in the manuscript that our main findings are robust to important methodological choices, and we detail where some minor results are not.
Randomization	Inventories are allocated into habitat clasess (floodplains or terra firme) based on the determination of the field collector. For species indicator analyses habitat classes were iteratively randomized to obtain a null distribution of species-habitat association strengths. But there was no experimental randomization approach.
Blinding	Not relevant. We did not use an experimental approach.

Did the study involve field work? ☒ Yes ☐ No

Field work, collection and transport

Field conditions	The Amazon is not an easy place to work. It is hot, humid, rainy, and field conditions are most often rustic.
Location	The Amazon region, northern South America.
Access & import/export	All collaborators have individually obtained the necessary permits for working and transportation of botanical vouchers.
Disturbance	Tree inventories have minimal impact for brief periods of time.

Reporting for specific materials, systems and methods

We require information from authors about some types of materials, experimental systems and methods used in many studies. Here, indicate whether each material, system or method listed is relevant to your study. If you are not sure if a list item applies to your research, read the appropriate section before selecting a response.

Materials & experimental systems

n/a	Involved in the study
☒	☐ Antibodies
☒	☐ Eukaryotic cell lines
☒	☐ Palaeontology and archaeology
☒	☐ Animals and other organisms
☒	☐ Clinical data
☒	☐ Dual use research of concern

Methods

n/a	Involved in the study
☒	☐ ChIP-seq
☒	☐ Flow cytometry
☒	☐ MRI-based neuroimaging
